# Investigation on Application Prospect of Refractories for Hydrogen Metallurgy: The Enlightenment from the Reaction between Commercial Brown Corundum and Hydrogen

**DOI:** 10.3390/ma15197022

**Published:** 2022-10-10

**Authors:** Shaofei Li, Ding Chen, Huazhi Gu, Ao Huang, Lvping Fu

**Affiliations:** The State Key Laboratory of Refractories and Metallurgy, Wuhan University of Science and Technology, Wuhan 430081, China

**Keywords:** Al_2_O_3_ based refractories, brown corundum, chemical stability, hydrogen metallurgy

## Abstract

Hydrogenous environments put forward new requirements to refractories for the hydrogen metallurgy field. The temperature and impurities in refractories played an important role in stability. A commercial brown corundum with many impurities was adopted as a raw material, thermodynamic calculations and reduction experiments of the brown corundum by high-purity hydrogen (99.99%) were accepted to investigate the stability of the oxides. The weight loss and mass fraction were tested to estimate the stability of the oxides in the brown corundum. XRD and SEM were used to analyze the mineral compositions and microstructures. The results showed that: the thermodynamic stability of the oxides in the brown corundum under high-purity hydrogen was in the order of Al_2_O_3_ > CaO > MgO > SiO_2_ > TiO_2_ > Fe_2_O_3_ at temperatures lower than 1400 °C. Obvious weight loss appeared after heating at 1400 °C for 8 h. The content of CaO did not decline after reduction even at 1800 °C, owing to the formation of hibonite (CaAl_12_O_19_), high-purity Al_2_O_3_ and CaAl_12_O_19_ -based refractories had the prospect for lining materials in the hydrogen metallurgy field, owing to their excellent chemical stability under hydrogenous environments.

## 1. Introduction

The process of iron ore reduction by carbon in iron and steel metallurgy has contributed the most greenhouse gas emissions in the whole process of iron and steel production. CO_2_ emissions from the steel industry have constituted a high proportion, equivalent to about 33.8% of industrial emissions [1]. The enhancement of clean energy technology and optimizing energy structures are crucial for carbon emissions reduction [2]. Therefore, environmentally friendly and economical iron ore reduction methods have become a hot spot in the exploration of an ideal process. Hydrogen metallurgy is a technology that partially or completely uses hydrogen to replace carbon as the reduction agent to reduce iron ore to obtain solid iron at the temperature range below the melting point of Fe, which has been very beneficial through energy savings and the reduction in CO_2_ emissions [3].

Refractories were indispensable key materials in the iron and steel industry, the hydrogenous environment has put forward new requirements due to the strong reducibility of hydrogen. Tso [4] has studied reactions between fused silica and hydrogen gas in the temperature range from 1200 °C to 1400 °C, the formation of SiO (g) and H_2_O (g) has caused the main weight loss. Herbell [5] has investigated the reactions between dry/wet hydrogen and some high-temperature ceramics including Al_2_O_3_, MgO, SiC, Si_3_N_4_ and mullite at 1400 °C, the increase in moisture provided the most benefits for stability, the Al_2_O_3_ ceramic performed excellently in stability. However, the reaction of Al_2_O_3_-based ceramics with hydrogen at the temperature range over 1400 °C needs further research.

Presently, blast furnace ironmaking adding hydrogen and direct reduction iron by hydrogen (DRI) are the two main hydrogen metallurgy methods [6,7], the temperature of the former process is about 1500 °C, the DRI is 750–1360 °C [8,9,10,11,12,13,14,15,16]. In addition, raising the temperature is the trend because a high temperature means high productivity in the hydrogen metallurgy process. So, the stability of Al_2_O_3_-based materials under a hydrogen atmosphere in a wide temperature range should be studied. Furthermore, the presence of impurities has played a significant role in the stability of the refractories and high-temperature ceramics under a hydrogen atmosphere.

Commercial corundum raw materials including brown corundum, white corundum, and tabular corundum are the main Al_2_O_3_-based refractory raw materials. In general, the content of Al_2_O_3_ in the latter two corundum raw materials is close to 99%. There are many impurities in brown corundum. In this paper, the commercial brown corundum was adopted as a raw material to react with the high-purity hydrogen in a wide temperature range from 600 °C to 1800 °C. The mass fractions of the oxides and mineral phase changes were tested to estimate the stability of the oxides in the brown corundum. The results have been beneficial for seeking stable refractories for the hydrogen metallurgy field.

## 2. Materials and Methods

**Figure 1:**
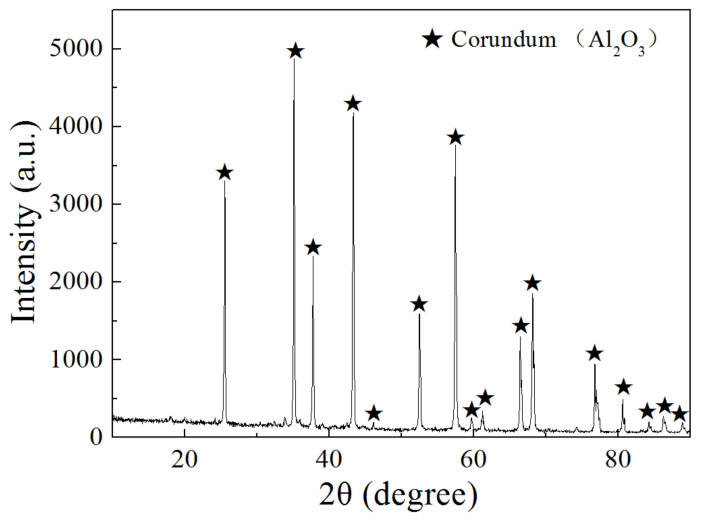
XRD pattern of the brown corundum raw material.

Commercial brown corundum powder (≤0.045 mm) was chosen as the raw material. The chemical compositions are shown in Table 1, the raw material consisted of 92.97 wt% of Al_2_O_3_ and some impurities including SiO_2_, CaO, TiO_2_ and MgO. The XRD pattern (Figure 1) showed the mineralogical compositions of the raw material, Al_2_O_3_ (corundum) was the main mineral phase. The peaks of the minerals related to the impurities are not shown in Figure 1, which might be caused by the following two reasons. First, the amount of such minerals containing impurities was too little. Second, impurities were distributed in the amorphous glass phases. High-purity hydrogen (99.99% of volume fraction) was adopted as the reductive agent.

The brown corundum was placed in molybdenum crucibles. The brown corundum raw material was dried at 110 °C for 24 h in a vacuum oven. The samples were ready for experiments after drying. Then the crucibles containing the brown corundum raw materials were put into a high temperature hydrogen furnace and heated to 600 °C, 700 °C, 800 °C, 1200 °C, 1400 °C, 1600 °C and 1800 °C for 8 h, respectively, under the high-purity hydrogen atmosphere. The heating rate was 3~6 °C/min, the flow rate of hydrogen was 2500 mL/min.

The masses of the brown corundum before and after heating were measured accurately. The weight loss of the brown corundum was evaluated as follows:

Weight loss = (M − M0)/M0(1)

Therein, M0 is the mass of a molybdenum crucible containing the brown corundum raw material before heating, M is the mass of a molybdenum crucible containing the brown corundum raw material after heating at different temperatures.

Then, the mass fractions of the Al_2_O_3_ and main impurities including SiO_2_, Fe_2_O_3_, CaO, TiO_x_, MgO in the brown corundum were measured via XRF (X-ray Fluorescence). The microstructures and mineralogical compositions were tested via SEM (SEM, JSM-6610, JEOL, Tokyo, Japan) and X-ray diffraction (XRD, X’ Pert Pro, Philips, The Netherlands).

## 3. Results and Discussion

### 3.1. Thermodynamic Calculations

The oxides in the brown corundum could react with the hydrogen, which would form gaseous products and result in weight loss. The equilibrium partial pressure of the gaseous products could be calculated. The reaction between SiO_2_ and H_2_ was taken as an example. Equation (2) showed the reaction, and the equilibrium partial pressures of SiO (g) and H_2_O (g) could be calculated according to Equation (3) under the hydrogen atmosphere [4]. For Equation (3), the pressure of H_2_ and the value of *P^θ^* were both 10^5^ Pa. The results were obtained and are shown in Table 2, the equilibrium partial pressures of SiO (g) and H_2_O (g) were both 2.7 Pa at 1200 °C, which would cause a slight weight loss of the brown corundum. At 1600 °C, the value was 345.2 Pa, which indicated serious degradation of SiO_2_ in the hydrogen furnace.

SiO_2_ (s) + H_2_ (g) = SiO (g) + H_2_O (g) (2)

(3) $$\ln\frac{\frac{P_{SiO}}{P^{\theta }}\cdot \frac{P_{H_{2}O}}{P^{\theta }}}{\frac{P_{H_{2}}}{P^{\theta }}}=\frac{\Delta_{r}G_{m}^{\theta }}{-RT}$$

The reaction conditions of the other oxides were calculated via the same method and the results are shown in Table 2.

Al_2_O_3_ was the most stable oxide in the brown corundum, the equilibrium partial pressures of Al (g) and H_2_O (g) were 2.0 × 10^−12^ Pa and 3.0 × 10^−12^ Pa at 1800 °C, such a small sum of gaseous products had a negligible effect on mass. CaO also performed well in stability when the temperature was lower than 1600 °C. The reduction process of TiO_2_ was complicated, a series of oxides of titanium would be generated [17,18], Ti_2_O_3_ was accepted as a reduction product for the calculation in Table 2. The TiO_x_ (x ≤ 2) was adopted to stand for oxides of titanium in the following contents. The thermodynamic stability of the oxides under high-purity hydrogen was in the order of Al_2_O_3_ > CaO > MgO > SiO_2_ > TiO_2_ > Fe_2_O_3_ at the temperature lower than 1400 °C, continuously increasing the temperature, the SiO_2_ performed worse in stability, compared with TiO_2_ and MgO. The Al_2_O_3_ could remain stable even at 1800 °C.

### 3.2. Weight Loss of Brown Corundum

**Figure 2:**
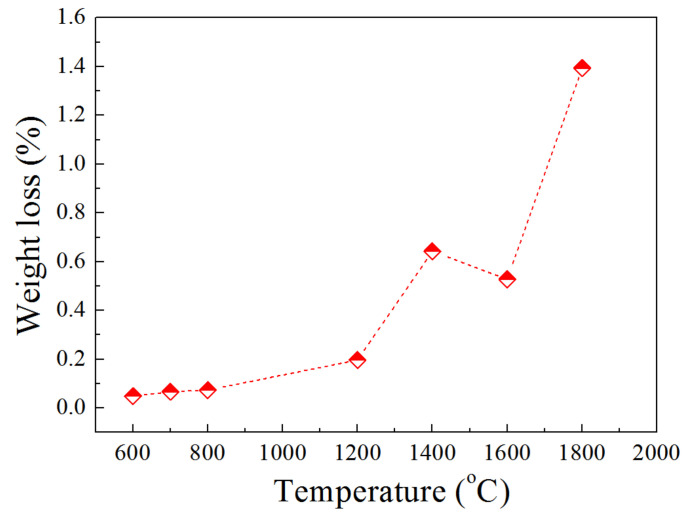
Weight loss of the brown corundum after reaction with high-purity hydrogen at different temperatures.

The Al_2_O_3_ in the brown corundum was stable under the H_2_ atmosphere according to Table 2. However, the brown corundum raw material consisted of impurities. Some impurities could react with H_2_ at elevated temperatures, which resulted in weight loss. The weight loss of the brown corundum after a high temperature hydrogen reduction is shown in Figure 2. The slight weight loss occurred when the temperature was increased to 1200 °C, which may be caused by the reduction in Fe_2_O_3_. Continuously raising the temperature to 1400 °C resulted in the increase in weight loss, the reduction in TiO_2_, MgO and SiO_2_ contributed a lot for weight loss.

**Figure 3:**
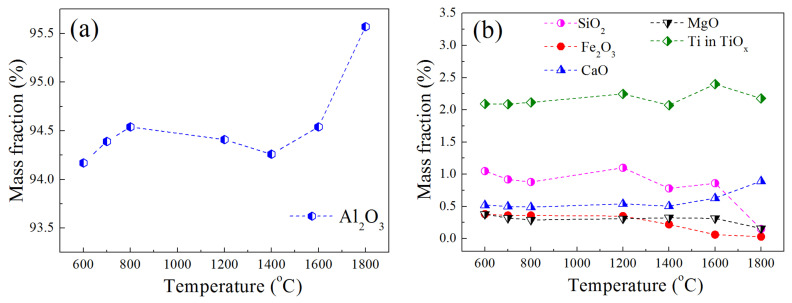
Mass fractions of different oxides in brown corundum after reaction with high-purity hydrogen at different temperatures: (**a**) Al_2_O_3_ and (**b**) impurity oxides.

The mass fractions of Al_2_O_3_ and impurities were tested and are shown in Figure 3. On the whole, the content of the Al_2_O_3_ presented an upward trend (shown in Figure 3a). Some impurities reacted with the H_2_, the gaseous products volatilized, while the Al_2_O_3_ had favorable stability in the high-temperature hydrogen furnace, which could have resulted in the increase in mass fraction of the Al_2_O_3_ and the decrease in mass fractions of the impurities. The mass fractions of the impurities after reduction for 8 h at different temperatures are shown in Figure 3b, the obvious mass fraction change occurred after rising up to 1400 °C, the mass fractions of TiO_x_ and CaO performed upward trends. The reaction between Fe_2_O_3_ and H_2_ could proceed at a low temperature according to Table 2, the contents of Fe_2_O_3_ decreased from 600 °C to 1200 °C, a continuously rising temperature accelerated the decline of Fe_2_O_3_. When the temperature was increased to 1400 °C, the mass fraction of SiO_2_ decreased obviously. On the whole, the Al_2_O_3_ was stable during heating under a hydrogen atmosphere up to 1800 °C. Furthermore, the CaO and TiO_x_ also performed excellently in stability during the experiments. TiO_2_ was easily reduced by H_2_ below 1600 °C, according to Table 2. However, the reduction product was solid phase TiO_x_ (x ≤ 2) with a high melting point. In the case of Ti_2_O_3_, the Ti_2_O_3_ had excellent stability under a hydrogen atmosphere. Equation (4) showed the reaction between the Ti_2_O_3_ and H_2_, the equilibrium partial pressure of H_2_O (g) could be calculated based on Equation (5), the value was 0.18 Pa at 1800 °C, which indicated a insignificant degradation of Ti_2_O_3_ under the hydrogen atmosphere at 1800 °C. The oxides of titanium performed an upward trend of mass fraction, with the increasing of temperature owing to the stability of TiO_x_.

Ti_2_O_3_ (s)+3H_2_ (g)=2Ti (s)+3H_2_O (g) (4)

(5) $$\ln\frac{\frac{P_{H_{2}O}}{P^{\theta }}}{\frac{P_{H_{2}}}{P^{\theta }}}=\frac{\Delta_{r}G_{m}^{\theta }}{-RT}$$

### 3.3. Phase Compositions of Brown Corundum after Reduction by Hydrogen

**Figure 4:**
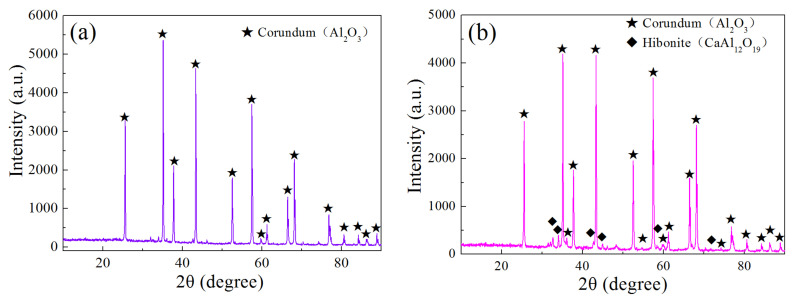
XRD patterns of brown corundum after reaction with high-purity hydrogen at different temperatures: (**a**) 1600 °C and (**b**) 1800 °C.

The mineral phases in brown corundum would change owing to the decrease in impurities. The main phases in the raw material were Al_2_O_3_ (corundum), based on Figure 1. After the reaction with hydrogen at 1600 °C for 8 h, no peaks containing impurities appeared. When increasing the reaction temperature to 1800 °C, a new mineralogical phase hibonite (CaAl_12_O_19_) was generated (shown in Figure 4b). According to Table 2, CaO could be reduced by H_2_ at 1800 °C, the pressure of the product H_2_O (g) was 60.9 Pa. The reason that the CaO content did not decline may be the formation of hibonite (CaAl_12_O_19_). As shown in Table 3, the pressures of Ca (g) and H_2_O (g) caused by the reaction between CaAl_12_O_19_ and H_2_ (g) were just 1.62 Pa at 1800 °C, the partial pressures of the gaseous products were much lower than the reaction of CaO and H_2_ (g), which meant a good stability of hibonite (CaAl_12_O_19_).

With the increasing of temperature, the contents of SiO_2_ decreased owing to the reduction reaction by hydrogen. The decrease in the SiO_2_ content in the amorphous glass phase was the concrete expression. SiO_2_ in the glass phase transferred to SiO (g), the volatilization of the gaseous products resulted in weight loss. Al_2_O_3_ (corundum) and CaAl_12_O_19_ (hibonite) were stable under the hydrogen atmosphere even at 1800 °C. Al_2_O_3_ and CaAl_12_O_19_ -based refractories were suitable for hydrogen metallurgy progress owing to their stability and availability of the raw materials.

### 3.4. Microstructure Evaluation of Brown Corundum after Reduction by Hydrogen

**Figure 5:**
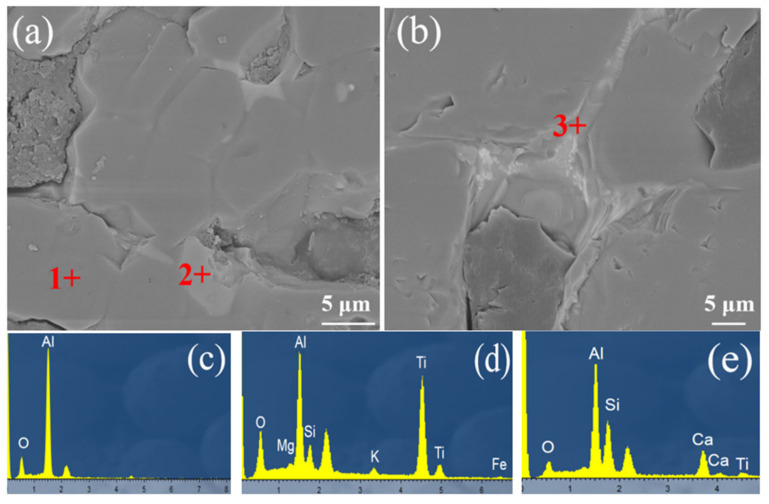
SEM photo of brown corundum materials (**a**) raw materials without reduction, (**b**) brown corundum after reduction by hydrogen at 1600 °C for 8 h, (**c**–**e**) are EDS of point 1, point 2 and point 3, respectively.

The microstructures of the samples were tested, the morphology and the EDS of the corundum materials are shown in Figure 5, the amorphous glass was distributed between the corundum particles, which consisted of the elements Ti, Al, Si, Mg, K, Fe and O, according to Figure 5d.

After heating at 1600 °C for 8 h in the hydrogen furnace, the compositions of the amorphous glass changed, the peaks of K, Fe and Mg disappeared. The elements Al, Ca, Ti, Si and O made up the amorphous glass based on Figure 5e. Compared with the raw materials, the elements Fe, Mg, and K decreased obviously owing to the reduction by hydrogen during heating at 1600 °C, the SiO_2_ impurities were still distributed between the corundum particles.

**Figure 6:**
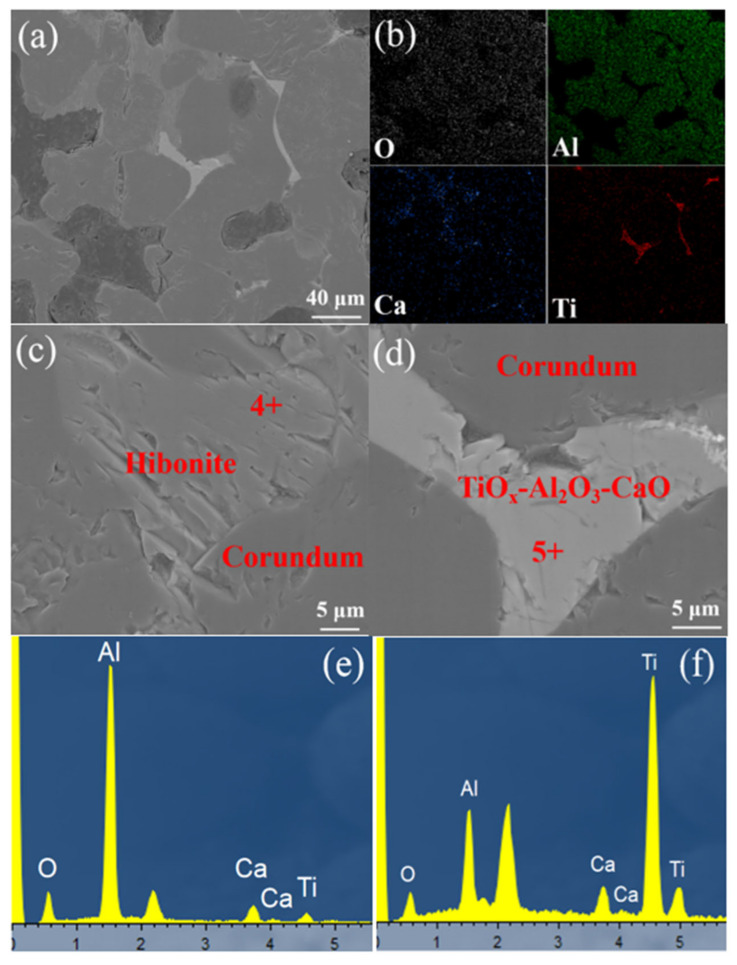
SEM photos of brown corundum after reduction by hydrogen at 1800 °C for 8 h: (**a**) microstructure, (**b**) EDS mapping of (**a**,**c**,**d**) are enlarged views of (**a**,**e**,**f**) are EDS of point 4 and point 5, respectively.

With continuous increasing of the temperature to 1800 °C, the phase compositions changed, according to Figure 6. The phases between corundum consisted of the elements Al, Ti, Ca and O. The element Si could not be detected, which indicated a violent reaction between SiO_2_ and hydrogen at 1800 °C. An amorphous glass of TiO_x_-Al_2_O_3_-CaO existed in the interface of the corundum particles. Furthermore, lamellar hibonite formed (Figure 6c), some TiO_x_ solubilized in the hibonite, according the result of EDS in point 4 (Figure 6e). The results indicated that the hibonite and corundum were the stable mineral phases under the hydrogen atmosphere, even at an elevated temperature of 1800 °C.

## 4. Conclusions

For the purpose of seeking suitable refractories for the hydrogen metallurgy field, thermodynamic calculations and reduction experiments of commercial brown corundum by high-purity hydrogen were adopted to investigate the stability of the oxides. The following results were obtained.

(1) The thermodynamic stability of the oxides under high-purity hydrogen was in the order of Al_2_O_3_ > CaO > MgO > SiO_2_ > TiO_2_ > Fe_2_O_3_ at the temperature lower than 1400 °C, when continuously increasing the temperature, the SiO_2_ performed worse in stability compared with TiO_2_ and MgO. The Al_2_O_3_ could remain stable even at 1800 °C. Obvious weight loss appeared when raising the temperature to 1400 °C. The reduction in Fe_2_O_3_, SiO_2_ and MgO contributed a lot to the weight loss.
(2) The pressures of Ca (g) and H_2_O (g) caused by the reaction between CaAl_12_O_19_ and H_2_ (g) was just 1.62 Pa at 1800 °C. The CaO contents in the brown corundum remained stable owing to the formation of hibonite (CaAl_12_O_19_).
(3) The corundum (Al_2_O_3_) and hibonite (CaAl_12_O_19_) performed excellently in stability under the high-purity hydrogen atmosphere, even at 1800 °C, which indicated that high-purity Al_2_O_3_ and CaAl_12_O_19_ -based refractories were suitable for lining materials in the hydrogen metallurgy field.

## Figures and Tables

**Table 1:** Chemical compositions of the brown corundum raw materials (wt%).

SiO_2_	Al_2_O_3_	Fe_2_O_3_	CaO	MgO	K_2_O	Na_2_O	TiO_2_
1.12	92.97	0.2	0.26	0.17	0.094	0.038	2.13

**Table 2:** Equilibrium partial pressures of gaseous products of reactions at different temperatures (Pa).

Reactions		1200 °C	1400 °C	1600 °C	1800 °C
SiO_2_ (s) + H_2_ (g) = SiO (g) + H_2_O (g)	P_SiO_	2.7	41.3	345.2	1884.3
	P_H2O_	2.7	41.3	345.2	1884.3
MgO (s) + H_2_ (g) = Mg (g) + H_2_O (g)	P_Mg_	2.2	23.2	146.2	639.4
	P_H2O_	2.2	23.2	146.2	639.4
Al_2_O_3_ (s) + 3H_2_ (g) = 2Al (g) + 3H_2_O (g)	P_Al_	9.3 × 10^−22^	7.0 × 10^−18^	7.5 × 10^−15^	2.0 × 10^−12^
	P_H2O_	1.4 × 10^−21^	1.0 × 10^−17^	1.1 × 10^−14^	3.0 × 10^−12^
CaO (s) + H_2_ (g) = Ca (g) + H_2_O (g)	P_Ca_	0.3	2.0	11.6	60.9
	P_H2O_	0.3	2.0	11.6	60.9
2TiO_2_ (s) + H_2_ (g) = Ti_2_O_3_ (s) + H_2_O (g)	P_H2O_	77.4	201.7	394.1	625.5
Fe_2_O_3_ (s) + 3H_2_ (g) = 2Fe + 3H_2_O (g)	P_H2O_	1.5 × 10^5^	1.3 × 10^5^	9.8 × 10^4^	7.3 × 10^4^

**Table 3:** Equilibrium partial pressures of gaseous products of reduction reactions of CaAl_12_O_19_ and CaO at different temperatures (Pa).

Reactions		1200 °C	1400 °C	1600 °C	1800 °C
CaAl_12_O_19_ (s) + H_2_ (g) = Ca (g) + H_2_O (g) + 6Al_2_O_3_	P_Ca_	3.6 × 10^−3^	4.7 × 10^−2^	0.35	1.62
	P_H2O_	3.6 × 10^−3^	4.7 × 10^−2^	0.35	1.62
CaO (s) + H_2_ (g) = Ca (g) + H_2_O (g)	P_Ca_	0.3	2.0	11.6	60.9
	P_H2O_	0.3	2.0	11.6	60.9

## Data Availability

Not applicable.
